# Treatment of Severe Obstructive Sleep Apnea With a Domestic Implantable Hypoglossal Nerve Stimulator: A Case Report

**DOI:** 10.1002/wjo2.70140

**Published:** 2026-08-17

**Authors:** Su‐Ru Liu, En‐Hui Zhou, Xin‐Yi Li, Hua‐Jun Xu, Jian Guan, Hong‐Liang Yi, Shan‐Kai Yin

**Affiliations:** ^1^ Department of Otorhinolaryngology Head and Neck Surgery Shanghai Sixth People's Hospital Affiliated to Shanghai Jiao Tong University School of Medicine, Shanghai Key Laboratory of Sleep Disordered Breathing, Otolaryngology Institute of Shanghai Jiao Tong University Shanghai China

**Keywords:** domestic device, hypoglossal nerve stimulator, obstructive sleep apnea, upper airway stimulation

## Abstract

**Background:**

Implantable hypoglossal nerve stimulation (HNS) is an established neuromodulatory therapy for obstructive sleep apnea (OSA) in patients intolerant to continuous positive airway pressure (CPAP). However, no domestically developed HNS system has been clinically implemented in China to date.

**Methods:**

We report the first clinical implantation of a novel, Chinese‐developed HNS system in a patient with severe OSA. The patient was a 39‐year‐old man with a 20‐year history of nocturnal snoring and apnea. Preoperative polysomnography (PSG) demonstrated an apnea–hypopnea index (AHI) of 46.3 events per hour and a lowest oxygen saturation of 83%. The device selectively activates the medial branch of the hypoglossal nerve, which induces genioglossus contraction and anterior tongue displacement, thereby stabilizing the upper airway during sleep.

**Results:**

The procedure was successfully performed. At short‐term follow‐up, the patient showed marked improvements: AHI decreased from 46.3 to 11.8 events/hour, LSaO_2_ increased from 83% to 86%, and the Epworth Sleepiness Scale Score improved from 1 to 0. No adverse events occurred.

**Conclusions:**

This first‑in‑human experience confirms that the domestic HNS system is feasible, safe, and clinically effective in patient with severe OSA who is intolerant to CPAP. These preliminary results support further clinical development and validation of this domestically manufactured HNS technology for broader application in China.

## Summary

- **Significant findings of the study**
  We report the first successful implantation of a Chinese‑developed implantable HNS system for severe OSA. The patient demonstrated marked symptomatic improvement with a favorable safety profile, and no adverse events occurred.
- **What this study adds**
  By establishing the clinical viability of a domestically developed HNS system, this study validates a safe, effective, and accessible alternative for CPAP‐intolerant patients. Beyond expanding therapeutic options, this innovation closes a critical technological gap and marks a strategic milestone in China's advancement toward clinical self‐reliance in neuromodulation therapy for sleep‐disordered breathing.

## 1 Case Presentation

A 39‐year‐old man was admitted on May 13, 2025, with a 20‐year history of snoring and nocturnal choking episodes. He reported a sensation of suffocation during sleep and habitual mouth breathing but denied other common symptoms, including nocturia, nightmares, palpitations, morning headache, excessive daytime sleepiness, fatigue, memory impairment, and difficulty concentrating. Polysomnography (PSG) performed on February 18, 2025 revealed a total sleep time (TST) of 505 min and predominantly obstructive respiratory events. The apnea–hypopnea index (AHI) was 71.9 events/hour, and the lowest oxygen saturation (LSaO_2_) was 78%. These findings were consistent with severe obstructive sleep apnea (OSA) accompanied by severe nocturnal hypoxemia. Continuous positive airway pressure (CPAP) was prescribed but was discontinued due to intolerance.

**Figure 1:**
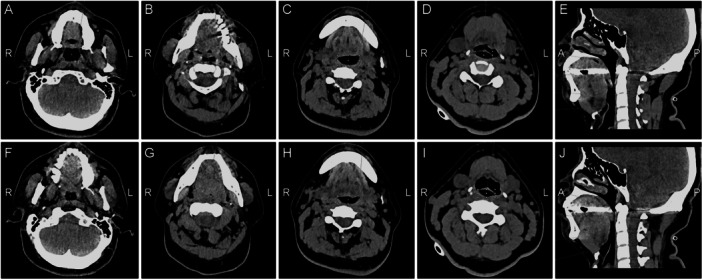
Preoperative upper airway computed tomography images during quiet breathing and deep inspiration. (A–E) Axial images during quiet breathing: (A) nasopharynx; (B) velopharynx; (C) retroglossal region; (D) retroepiglottic region; and (E) sagittal segmentation. (F–J) Axial images during deep inspiration: (F) nasopharynx; (G) velopharynx; (H) retroglossal region; (I) retroepiglottic region; and (J) sagittal segmentation.

The patient had a 3‐year history of hypertension and hyperuricemia, but no other chronic medical conditions. He had no history of smoking, alcohol consumption, or known family history of hereditary disease. Physical examination revealed a body mass index of 25.9 kg/m^2^, bilateral tonsillar hypertrophy (Grade II), tongue position Grade III, Friedman Stage III, lateral pharyngeal wall narrowing score of 2, and suboptimal epiglottic exposure on indirect laryngoscopy. Upper airway computed tomography demonstrated narrowing of the velopharyngeal region during deep inspiration (Figure 1). The Epworth Sleepiness Scale (ESS) Score was 1. Based on the clinical history and physical examinations, the preliminary diagnoses were severe OSA, hypertension, and hyperuricemia.

**Figure 2:**
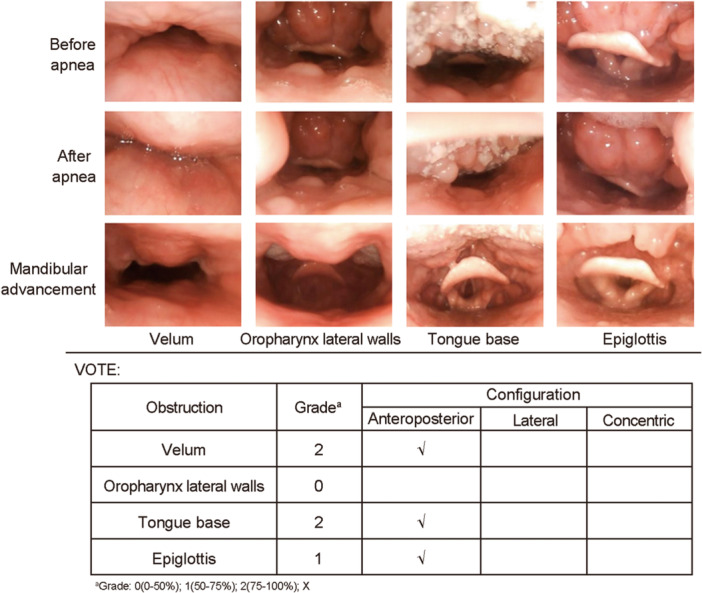
Preoperative drug‐induced sleep endoscopy findings.

PSG on the night of admission showed: TST 496.5 min, sleep efficiency 93.2%, predominantly obstructive respiratory events, AHI 46.3 events/hour, and LSaO_2_ 83%, indicating severe OSA with moderate hypoxemia. Drug‐induced sleep endoscopy (DISE) demonstrated Grade 2 anteroposterior collapse at the soft palate level and at the tongue base, with no clinically significant collapse of the oropharyngeal lateral walls. Grade 1 secondary anteroposterior collapse was noted at the epiglottic level. The total VOTE score was 5 (Figure 2), indicating multilevel airway collapse without complete concentric collapse—favorable anatomy for hypoglossal nerve stimulation (HNS).

## 2 Surgical Procedure and Outcomes

**Figure 3:**
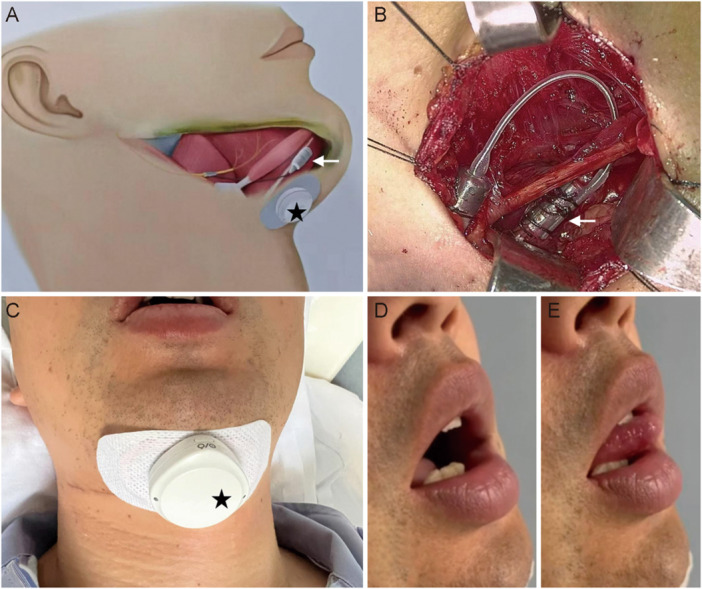
Implantation and device activation of hypoglossal nerve stimulation. (A) Illustration of the implantation procedure; (B) intraoperative photograph; (C) postoperative photograph; (D) tongue position before stimulation; and (E) tongue position after stimulation. The white arrow indicates the receiving device. The black star indicates the external transmitter.

After surgical contraindications were excluded, implantation of an HNS system was performed under general anesthesia on May 15, 2025. The key procedural steps were as follows (Figure 3A,B): (1) placement of intraoperative neuromonitoring electrodes on the right lateral tongue and the right genioglossus muscle in the floor of the mouth; (2) creation of a skin incision approximately 2.0 cm inferior to the right mandibular margin, followed by dissection through the subcutaneous tissue to expose the submandibular gland and identification of the hypoglossal nerve at its inferior border; (3) dissection and localization of the medial branch of the hypoglossal nerve using a nerve monitoring probe; (4) fixation of a cuff electrode around the medial branch of the hypoglossal nerve and placement of the receiver unit in the submental region; and (5) performance of intraoperative stimulation using an external transmitter to confirm adequate tongue protrusion. The patient was extubated after recovery from anesthesia and transferred to the ward in stable condition. No postoperative complications—including wound infection, tongue numbness, or tongue deviation—were observed. Device activation and parameter titration were performed 1 month after implantation (Figure 3C–E). Follow‐up PSG performed 1 month after activation (2 months postoperatively) demonstrated a TST of 504 min, sleep efficiency of 96.6%, predominantly obstructive respiratory events, an AHI of 11.8 events/hour (a 74.5% reduction from baseline), and an LSaO_2_ of 86%, consistent with mild OSA and mild hypoxemia. The ESS score improved to 0, indicating a marked therapeutic response. At the 4‐month follow‐up, the patient reported good adherence to HNS therapy, using the device an average of more than 5 nights per week. PSG showed an AHI of 27.6 events/hour and LSaO_2_ of 84%, with ESS score of 0. The patient reported high subjective satisfaction, and noted no tongue discomfort, swallowing difficulty, or stimulation‐induced awakening during sleep. No adverse events have been reported to date, and the patient continues to be followed.

## 3 Discussion

HNS is a neuromodulatory therapy that activates the upper airway dilator muscles—principally the genioglossus—via an implantable system. This stimulation protrudes the tongue and enlarges the retropalatal, retroglossal, and retroepiglottic spaces, thereby opening the airway and improving apnea and hypoxemia in patients with OSA [1]. Currently, the Inspire Medical Systems is the only device approved by the FDA for unilateral HNS in the treatment of OSA. The system comprises a cuff electrode, an implantable pulse generator, and a respiratory sensing pressure lead, which enable stimulation synchronized with inspiration [2].

Since receiving FDA approval in 2014, multiple prospective clinical studies have demonstrated the efficacy and durability of HNS therapy for the management of OSA. As an innovative neuromodulation strategy, HNS provides substantial clinical benefit with relatively few adverse effects and high patient adherence, offering a valuable alternative for patients who are intolerant to CPAP [3, 4, 5]. A recent meta‐analysis including 44 original studies (*n* = 8670) with up to 3 years of follow‐up demonstrated significant reductions in AHI, oxygen desaturation index, and ESS scores, with overall treatment response rates ranging from 61% to 96% [6]. With growing clinical experience, the FDA has expanded the upper AHI eligibility criterion (from 50 to 100 events/hour) and the BMI limit (from 32 to 40 kg/m^2^), and has approved the therapy for patients aged ≥ 13 years with Down syndrome. These updates highlight the broad therapeutic potential of HNS. The technique has now been incorporated into OSA treatment guidelines, particularly for patients with moderate‐to‐severe OSA who are intolerant to CPAP [6, 7]. Inclusion criteria for this study were: (1) a diagnosis of moderate‐to‐severe OSA; (2) age between 18 and 65 years; (3) tonsillar hypertrophy of Grade I to II. Exclusion criteria were: (1) comorbidity with other sleep disorders or severe systematic diseases; (2) previous surgery for OSA; (3) complete concentric collapse of the velopharynx on DISE; (4) pregnancy; (5) participation in another clinical trail; and (6) inability to provide informed consent or comply with study procedures.

Despite these advantages, HNS systems have certain limitations and can entail procedure‐related adverse events, such as incision pain, postoperative infection, and transient tongue weakness (typically resolving within weeks).

The domestically developed HNS system used in this case exhibits performance metrics comparable to those of leading international devices. This system incorporates several key technological innovations that distinguish it from existing products, including the Inspire system (Inspire Medical Systems) and the Genio system (Nyxoah), as summarized in Table 1.

**Table 1:** Comparison between domestic and foreign hypoglossal nerve stimulation (HNS) systems.

Dimension	Domestic HNS	Inspire HNS	Genio HNS
Stimulation mode	Unilateral stimulation	Unilateral stimulation	Bilateral stimulation
Implantation method	Single incision	Dual incision	Single incision
Driving mode	Respiratory cycle‐dependent stimulation	Respiratory cycle‐dependent stimulation	Time cycle‐dependent stimulation
Compatibility	1.5 T and 3.0 T MRI	1.5 T MRI	1.5 T and 3.0 T MRI
Battery	Wireless power, battery‐free implantable stimulator	Surgical replacement, battery‐powered implantable stimulator	Wireless power, battery‐free implantable stimulator
Transmission mode	Wireless in body	Wire in body	Wireless in body

In this first reported case of HNS implantation in China, the patient demonstrated significant postoperative improvements in both objective polysomnographic parameters and clinical symptoms. However, the observed increase in the AHI at 4 months—from 11.8 to 27.6 events/hour—warrants discussion. Several factors may contribute to this change, including neuromuscular adaptation with consequent reduction in stimulation efficiency, potential weight gain, and so forth. Long‐term follow‐up is ongoing.

Several limitations should be acknowledged. First, the single‐case nature of this report precludes statistical inference and broad generalization. Second, the limited follow‐up duration may not capture late‐phase changes in AHI or long‐term device‐related complications. Third, potential selection bias limits generalizability, as the patient met specific favorable anatomical and clinical criteria for HNS candidacy. Future larger‐scale, longer‐term studies are warranted to confirm these preliminary observations.

The successful implementation of this procedure represents a milestone in the treatment of OSA in China, addressing a critical clinical gap and laying the foundation for broader clinical adoption and industrial development. Through continued multidisciplinary collaboration between clinicians and engineers, further technological refinement and clinical validation will likely enhance patient outcomes and advance the field of sleep medicine.

## Author Contributions

**Shan‐Kai Yin** and **Hong‐Liang Yi:** conceptualization. **Su‐Ru Liu** and **En‐Hui Zhou:** data curation, writing – original draft preparation. **En‐Hui Zhou** and **Xin‐Yi Li:** visualization, investigation. **Hong‐Liang Yi:** supervision. **Su‐Ru Liu**, **Hua‐Jun Xu**, and **Jian Guan:** writing – reviewing and editing. All authors commented on previous versions of the manuscript. All authors read and approved the final manuscript.

## Ethics Statement

This study was approved by the Ethics Committee of Shanghai Sixth People's Hospital Affiliated to Shanghai Jiao Tong University School of Medicine (Approval No: 2028‐YS‐553). The research was conducted in accordance with the Declaration of Helsinki. All patients provided written informed consent.

## Conflicts of Interest

Professor Shan‐Kai Yin is a member of *World Journal of Otorhinolaryngology – Head & Neck Surgery* (WJOHNS) editorial board and is not involved in the peer review process of this article. The other authors declare no conflicts of interest.

## Data Availability

The datasets supporting the conclusions of this article are included within the article and its additional files.
